# Global breast cancer incidence, mortality, and survival among indigenous women: A systematic review and meta-analysis

**DOI:** 10.1016/j.breast.2026.104742

**Published:** 2026-02-26

**Authors:** Halijah Brewster, Tsegaw Amare Baykeda, Sewunet Admasu Belachew, Valerie McCormack, Miranda Fidler-Benaoudia, Gail Garvey

**Affiliations:** a First Nations Cancer and Wellbeing Research Program, School of Public Health, The University of Queensland, Brisbane, Australia; b International Agency for Research on Cancer, Lyon, France; c University of Calgary, Calgary, Canada

**Keywords:** Breast cancer, Indigenous women, Incidence, Mortality, Systematic review

## Abstract

Breast cancer is the most common cancer diagnosed in women worldwide. Our understanding of the burden of breast cancer among Indigenous women remains limited due to limited availability of Indigenous data in global statistical databases. To address this concern, we systematically reviewed existing evidence of breast cancer incidence, mortality, and survival among Indigenous women by searching PubMed, Web of Science, CINAHL, and Embase using the terms “breast cancer,” “incidence,” “mortality,” “survival,” and “Indigenous peoples.” A random-effects meta-analysis was performed to estimate the pooled adjusted hazard ratio (aHR). Overall, 61 studies from the United States (35), Australia (9), New Zealand (8), Canada (5), and Brazil (2), as well as one each from Peru and Colombia, were included in our analysis. Our findings revealed age-adjusted incidence rates of breast cancer ranging from 19.0 to 165.2 per 100,000 Indigenous women, compared to 21.5 to 190.4 per 100,000 non-Indigenous women, accompanied by a 32% higher hazard of mortality (aHR = 1.3; 95% confidence interval: 1.2–1.5). Pooled aHRs were 1.5 in Oceania and 1.1 in North America, and 1.4 and 1.2 for the years 2013–2017 and 2018–2023, respectively. Despite improvements over time and a lower overall incidence, Indigenous women have a higher hazard of mortality from breast cancer than their non-Indigenous counterparts. Continued efforts to enhance early detection and healthcare access are essential to improve the clinical outcomes of breast cancer among Indigenous women. Additional research targeting Africa, Asia, and Latin America will improve our understanding of the overall global burden of breast cancer.

## Highlights

• First global meta-analysis of breast cancer in Indigenous women.
• Indigenous women have 32% higher mortality hazard (pooled aHR = 1.3).
• Lower overall incidence but poorer survival than non-Indigenous women.
• Mortality disparities persist across regions and time periods.

## Abbreviations

aHR -: adjusted hazard ratio
ASIR -: Age standardized incidence rate
CI -: confidence interval
DL -: DerSimonian and Laird
LCI -: lower confidence interval
NOS -: Newcastle-Ottawa Scale
NZ -: New Zealand
PRISMA -: Preferred Reporting Items for Systematic Reviews and Meta-Analysis
UCI -: upper confidence interval
USA -: United States of America

## 1 Introduction

Breast cancer is the most frequently diagnosed neoplastic disease and a leading cause of death among women globally [1]. The incidence of breast cancer continues to increase, with an age-adjusted global incidence rate of 46.8/100,000 reported in 2022 [2]. Breast cancer incidence rates are reported to be higher in high-income countries (76 per 100,000) than in low-income countries (34 per 100,000) [1]. Kim et al. [3] revealed four-fold variations in reported age-adjusted incidence rates between sub-regions, with incidence rates highest in Australia and New Zealand at 100.3 per 100,000 (95% confidence interval [CI]: 98.8–101.8) and lowest in South-central Asia (26.7 per 100,000, 95% CI: 26.5–26.9). In recent years, with improved cancer treatments, population screening programs, and earlier-stage diagnosis across all ages, breast cancer mortality rates have declined [4,5]. However, these improvements are not universal, with inequities apparent within individual countries [4,6] and across socioeconomic groups [7].

Indigenous peoples, comprising 6.2% of the world's population across 90 countries [8], have traditions, languages, social, cultural, economic, and political characteristics that are distinct from those of the dominant population [8]. Despite this rich diversity, many Indigenous peoples often experience health inequalities, including poorer outcomes and lower life expectancy compared to non-Indigenous populations in the same region [9]. In recent years, cancer has emerged as a significant health priority for Indigenous populations worldwide, driven by substantial disparities in cancer burden between Indigenous and non-Indigenous populations within and across countries [10,11].

Several countries with well-resourced cancer registries, such as Australia and New Zealand, have reported cancer as a leading cause of death among their Indigenous populations [12], with poorer outcomes and lower survival rates compared to non-Indigenous people in the same setting [12]. For example, in New Zealand, Maori women with breast cancer have an adjusted mortality ratio of 1:76 compared with their non-Maori counterparts [13]. Similarly, Indigenous women in Australia are 1.2 times more likely to die from breast cancer, and have a five-year survival rate of 81% compared to 90% for non-Indigenous women with breast cancer [12].

Factors associated with disparities in breast cancer mortality include race/ethnicity, socioeconomic status, molecular subtype, access to health services and surgical treatment, higher comorbidity rates, age at diagnosis, and a greater likelihood of advanced tumour stages at presentation [6,[13], [14], [15], [16], [17], [18], [19], [20], [21]]. Additionally, lower rates of participation in breast cancer screening among Indigenous compared to non-Indigenous women further exacerbate these disparities [12,13]. These observations underscore the critical need for robust and accurate data collection to improve our understanding of the full extent of the burden of breast cancer among Indigenous populations, which can be used to guide targeted interventions.

While there is existing data on breast cancer incidence, mortality, and survival among Indigenous women in some countries, comprehensive global epidemiological information on the burden of breast cancer among Indigenous populations are lacking. Currently, availability and quality of relevant data preclude accurate or comprehensive global assessments of breast cancer statistics among Indigenous populations globally [22]. This systematic review was undertaken to review and pool epidemiological data from multiple peer-reviewed publications to gather evidence of the current state of the burden of breast cancer among Indigenous women globally to inform future policies and develop targeted strategies to improve breast cancer outcomes among Indigenous women.

## 2 Methods and materials

This review was conducted according to the Preferred Reporting Items for Systematic Reviews and Meta-Analysis (PRISMA) guidelines (Table S1) [23]. The protocol of this systematic review is registered in PROSPERO (Identifier: CRD420251108805).

### 2.1 Indigenous terminology

This review uses the term “Indigenous peoples” to refer to groups of people who identify as such, including American Indian, Alaska Native, Native Hawaiian, Maori, First Nations, Inuit, Métis, Aboriginal, and Torres Strait Islander peoples, as well as others who identified themselves with this term in published studies. We acknowledge that not all Indigenous peoples worldwide use the same terminology to identify themselves. The full list of Indigenous terms used in the search strategies is provided in a supplementary file (Table S2). Additionally, we use the term “non-Indigenous peoples” as a comparator to refer to any individuals not identified as Indigenous in these studies. Consistent with previous studies [24], we categorized Whites, European New Zealanders, non-Indigenous Australians, and other groups as “non-Indigenous peoples.”

### 2.2 Search strategies and eligibility criteria

A systematic search was conducted using international databases, including Medline via PubMed, Web of Science, CINAHL, and Embase, all provided by Elsevier. The search included keywords such as “breast cancer,” “incidence,” “mortality,” “survival,” and “Indigenous peoples.” Our search was conducted on June 9, 2025, to identify studies published in English since 2013. The keywords were combined using standard Boolean operators (AND/OR) and Medical Subject Headings (MeSH) in PubMed, as well as Emtree terms in Embase. To refine the search terms for different databases, we used the Systematic Review Accelerator [25] (Table S2). We included quantitative studies of Indigenous women with breast cancer and studies comparing Indigenous and non-Indigenous women with breast cancer that reported at least one of three main outcomes: incidence rates, mortality rates, and survival rates. Commentaries, editorials, reviews, randomized controlled trials, protocols, research dissertations, qualitative studies, animal studies, case studies, and conference abstracts were excluded.

### 2.3 Study selection and quality assessment

The studies identified in the systematic search were imported into EndNote, and, after duplicates were removed, they were exported to Covidence software. Two reviewers, TAB (a non-Indigenous researcher) and HB (an Aboriginal researcher), screened the article titles, abstracts, and full texts according to predefined eligibility criteria. Any disagreements over inclusions were addressed through discussion and, if necessary, resolved by a third author (SAB).

The Newcastle-Ottawa Scale (NOS) was used to assess the quality of these studies. This scale uses a star rating system to assess a study based on three perspectives: selection, comparability, and outcome; each with four, one, and three components, respectively. Given the highest possible score of nine stars, each study received a maximum of one star for each component in the selection and outcome sections, and up to two stars for the comparability section. Study quality was rated from 0 to 9 stars and was ranked as follows: studies with 6–9 stars were considered good, those with 3–5 stars were considered fair, and those with 0–2 stars were classified as poor [26].

### 2.4 Data extraction and synthesis

The two reviewers independently extracted data by using a pre-defined data extraction form in Microsoft Excel 2010. Study characteristics, including author; publication year; study country; study level; study design; study period; age group; Indigenous peoples; number of Indigenous cases; non-Indigenous peoples; number of non-Indigenous cases; and data source; as well as key findings, such as incidence, mortality, and survival rates for both Indigenous and non-Indigenous women were collected from these publications.

All data analyses were conducted using Stata 18 software. A descriptive report of the study characteristics, as well as the incidence, mortality, and survival rates, was produced for narrative synthesis. Publication bias was evaluated through visual inspection of funnel plots and the *p*-value from Egger's test. Heterogeneity among studies was assessed using Cochran's Q test (p < 0.05) and *I*^2^ statistics. Given the significant heterogeneity among studies, we employed the DerSimonian and Laird random-effects meta-analysis to calculate the pooled effect. The adjusted hazard ratio (aHR) for breast cancer mortality in Indigenous women compared to non-Indigenous women was pooled to assess the pooled hazard of breast cancer mortality among Indigenous women. Because some articles did not report CIs and standard errors, we calculated the standard error by using the binomial distribution for the studies included in the meta-analysis. We also performed subgroup meta-analyses by country, publication year, and age group, as well as sensitivity analyses in which we omitted one study at a time to assess its effect on the pooled estimate. We conducted a meta-regression to examine the association between study characteristics, such as age group, publication year, and number of breast cancer cases, with the pooled log HR of breast cancer mortality among Indigenous peoples.

## 3 Results

### 3.1 Study selection

**Fig. 1:**
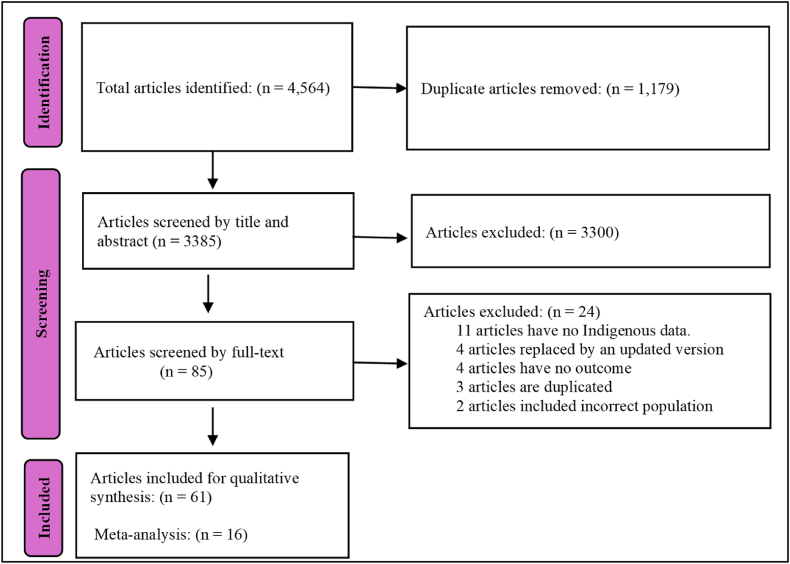
PRISMA flow diagram of study selection.

Our systematic search yielded 4564 studies, of which 1179 were identified as duplicates. After screening the titles and abstracts, we identified 85 studies that were eligible for full-text review. Of these, 61 studies were subsequently included in our qualitative synthesis. Sixteen of these 61 studies were included in the meta-analysis (Fig. 1).

### 3.2 Study characteristics

Of the 61 studies included in our review, 60 were retrospective cohort studies and one was a case-control study. Fifty-five studies used cancer registries as their primary data source, while six [14,[27], [28], [29], [30]] used alternative sources. Nineteen studies involved participants of all ages, 30 studies covered participants from young adolescence to adulthood, three studies were limited to adults under 50 years of age, and eight studies did not specify the age group. The studies were conducted in the United States (n = 35), Australia (n = 9), New Zealand (n = 8), Canada (n = 5), and three countries in South America (n = 4). Thirty-six studies were conducted at the national level, while 25 studies were conducted at the state level, with data collection periods ranging from 1973 to 2022.

A total of 102,490 breast cancer cases were reported among Indigenous women, ranging from three in Brazil [31] to 20,325 cases in the United States [32]. By contrast, 15,060,455 breast cancer cases were reported among non-Indigenous women, ranging from 67 in New Zealand [33] to 3,341,855 in the United States [32] (Table 1). The 61 studies were evaluated for quality using the Newcastle-Ottawa Scale and achieved scores ranging from seven to nine stars, with more than half (n = 37) scoring a full nine stars. In total, 59 studies met the selection criteria, 58 studies fulfilled the comparability criteria, and 41 studies met the outcome criteria (Table S3).

**Table 1:** Characteristics of included studies, by country and publication year (2013–2025).

Author	Study setting	Study period	Data source	Age group (years)	Cases in Indigenous women	Cases in non-Indigenous women	Outcomes						
							Indigenous women				Non-Indigenous women		
							Population-level		Breast cancer patients		Population-level		Breast cancer patients
							Incidence	Mortality	Mortality risk^a^	Survival	Incidence	Mortality	Survival
**United States of America**													
De Grubb, 2013 [34]	National	1999–2009	CDC NPCR	≥35	NR	NR	NR	AAMR = 29.60 (28.10–31.10)	NR	NR	NR	AAMR = 47.50 (47.30–47.60)	NR
Liu, 2013 [35]	National	2002–2008	SEER-13	NR	1284	228,639	AAIR = 136.80 (129.40–144.50)	NR	NR	NR	AAIR = 136.60 (136.00–137.20)	NR	NR
Tannenbaum, 2013 [17]	Florida	1996–2007	Florida Cancer Registry	≥18	60	115,506	NR	NR	aHR = 1.19 (0.81–1.73)	5-yr CrSurv = 75.30	NR	NR	5-yr CrSurv = 74.50
Campbell, 2014 [36]	Oklahoma	2005–2009	Oklahoma Cancer Registry	All ages	973	10,818	AAIR = 140.50	NR	NR	NR	AAIR = 121.50	NR	NR
Lee, 2014 [20]	Florida	1996–2007	Florida Cancer Registry	≥18	38	101,517	NR	NR	aHR = 1.52 (1.05–2.20)	NR	NR	NR	NR
Roen, 2014 [37]	Michigan	2004	Michigan Cancer Registry	≥30	19	6106	AAIR = 64.80 (33.60–96.00)	NR	NR	NR	AAIR = 124.90 (121.80–128.10)	NR	NR
White, 2014 [38]	Northern Plains, Alaska, Southern Plains, Southwest, West coast, East coast	1999–2009	SEER	All ages	1970	NR	AAIR = 100.0 (97.50–102.50)	AAMR = 22.30 (21.10–23.60)	NR	NR	AAIR = 131.30 (130.90–131.70)	AAMR = 24.10 (23.90 -24.20)	NR
Watanabe- Galloway, 2015 [39]	Nebraska, North Dakota, and South Dakota	2002–2009	Nebraska, North Dakota, and South Dakota Cancer Registry	≥19	409	20,039	AAIR = 46.70 (39.10–54.30)	NR	NR	NR	AAIR = 43.40 (42.30–44.50)	NR	NR
Campbell, 2016 [40]	Oklahoma	1997–2008	Oklahoma Cancer Registry	All ages	2425	30,317	NR	NR	NR	5-yr CrSurv = 80.20 (78.50 -81.70)	NR	NR	5-yr CrSurv = 79.40 (79.00 -79.90)
Emerson, 2017 [41]	Northern California [Kaiser Permanente]	1997–2015	Kaiser Permanente Northern California Cancer Registry	All ages	179	25,548	NR	NR	aHR = 1.31 (0.88 -1.94)	PropAlive = 69.30	NR	NR	PropAlive = 70.80
Khan, 2017 [42]	California, Connecticut, Georgia, Hawaii, Iowa, and New Mexico	1973–2011	SEER	≥20	87	9343	NR	NR	NR	Survival time months (SD) = 9.10 (7.70)	NR	NR	Survival time months (SD) = 9.27 (6.58)
Nash, 2018 [43]	Alaska and Washington	1992–2013	Alaska Native Tumor Registry	All ages	996	NR	NR	NR	NR	5-yr CrSurv = 89.60	NR	NR	NR
Shoemaker, 2018 [44]	National	2004–2013	SEER	20–49	2628	297,852	AAIR = 51.2	NR	NR	NR	AAIR = 73.0 (72.30–73.50)	NR	NR
Hill, 2019 [45]	National	2005–2009	SEER	All ages	1340	202,216	NR	NR	HR = 1.70 (1.30–2.30)	5-yr CrSurv = 86.0	NR	NR	5-yr CrSurv = 89.0
Melkonian, 2019 [46]	National	2010–2015	SEER and CDC NPCR	NR	NR	NR	AAIR = 112.5	NR	NR	NR	AAIR = 129.90	NR	NR
Nash, 2019 [47]	Alaska	2004–2014	SEER	All ages	697	181,676	AAIR = 145.0 (130.40–159.50)	NR	NR	NR	AAIR = 133.0 (132.2–133.8)	NR	NR
Gopalani, 2020 [48]	National	1999–2015	CDC NPCR	≥25	17,503	2,851,031	AAIR = 72.7 (71.60–73.80)	NR	NR	NR	AAIR = 130.40 (130.30–130.60	NR	NR
Longacre, 2020 [49]	National	2000–2015	SEER	≥18	1016	627,926	NR	NR	aHR = 1.30 (1.03–1.63)	NR	NR	NR	NR
Hendrick, 2021 [50]	National	2014–2018	SEER 21	≤50	1336	246,943	IR/100,000 = 28.6	MR/100,000 = 3.0	NR	NR	IR/100,000 = 49.20	MR /100,000 = 4.00	NR
Melkonian, 2021 [51]	Southern Plains	2012–2016	SEER and CDC	NR	1337	11,905	AAIR = 158.70	NR	NR	NR	AAIR = 119.0	NR	NR
Cronin, 2022 [52]	National	2001–2019	North American Association of Central Cancer Registries	NR	NR	NR	AAIR = 119.10 (115.60–122.80)	AAMR = 17.80 (16.50–19.20)	NR	NR	AAIR = 134.70 (134.40–135.00)	AAMR = 19.90 (19.80–20.00)	NR
Du, 2022 [53]	National	2000–2018	SEER 18	≥20	5829	802,790	AAIR = 128.8 (125.40–132.30)	NR	NR	NR	AAIR = 190.40 (190.00–190.80)	NR	NR
Ellington, 2022 [32]	National	1999–2018	SEER and CDC NPCR	≥20	20,325	3,341,855	AAIR = 127.30	NR	NR	NR	AAIR = 186.5	NR	NR
Giaquinto, 2022 [4]	National Except Nevada	2015–2019	SEER 17 and CDC NPCR	≥20	NR	NR	AAIR = 111.30	AAMR = 20.50	NR	NR	AAIR = 133.70	AAMR = 19.70	NR
Primm, 2022 [54]	National	2000–2017	SEER 18	≥20	3829	532,147	NR	NR	aHR = 1.10 (1.00–1.30)	5-yr CrSurv = 95.30	NR	NR	5-yr CrSurv = 95.70
Taparra, 2022 [55]	National	2004–2017	National Cancer Database	≥18	4955	1,832,693	NR	NR	aHR = 1.09 (1.00–1.20)	NR	NR	NR	NR
Du, 2023 [56]	National	2010–2019	SEER 18	≥20	3428	385,782	AAIR = 131.40 (126.90–136.00)	NR	NR	NR	AAIR = 176.90 (176.40–177.50)	NR	NR
Ellington, 2023 [21]	National	1999–2020	CDC NPCR	≥25	3679	700,704	NR	AAMR = 13.70	NR	NR	NR	AAMR = 19.40	NR
Gaba, 2023 [15]	National	2004–2015	National Cancer Database	≥18	6866	1,987,324	NR	NR	aHR = 1.00 (0.90–1.20)	NR	NR	NR	NR
Ihenacho, 2023 [57]	Hawaii	1990–2014	SEER and Hawaiʻi Tumor Registry	≤50	196	237	AAIR = 33.20 (28.70–38.10)	NR	NR	NR	AAIR = 39.80 (34.80–45.10)	NR	NR
Melkonian, 2023 [58]	Northern Plains, Alaska, Southern Plains, Pacific Coast, East, and Southwest	1999–2019	SEER and CDC NPCR	15–39	986	30,948	AAIR = 19.0 (17.90–20.30)	NR	NR	NR	AAIR = 21.50	NR	NR
Haque, 2023 [59]	National	2018-2020	National Centre for Health Statistics (NCHS)	All ages	NR	NR	NR	AAMR = 24.80	NR	NR	NR	AAMR = 27.40	NR
Islami, 2023 [60]	National	2016-2020	SEER and CDC NPCR	All ages	NR	NR	AAIR = 106.80	AAMR = 20.50	NR	5-yr CrSurv = 85.00%	AAIR = 142.90	AAMR = 19.70	5-yr CrSurv = 92.00%
Xu, 2024 [61]	National	2000-2019	SEER	20-49	1485	126,960	AAIR = 52.60	NR	NR	NR	AAIR = 69.70	NR	NR
Monticciolo, 2025 [62]	National	2015-2022	SEER-22	All ages	NR	NR	NR	APC = −1.53 (−5.56 to 0.39)	NR	NR	APC = −1.05 (−1.18 to −0.87)	NR	NR
**Australia**													
Supramaniam, 2014 [18]	NSW	2001–2007	NSW Cancer Registry	≥18	288	27,562	NR	NR	aHR = 1.30 (0.94–1.75)	5-yr CrSurv = 82.30% (76.80% – 87.10%)	NR	NR	5-yr CrSurv = 88.40% (88.00% – 88.80%)
Baade, 2016 [63]	QLD	2007–2012	QLD Cancer Registry	20–89	208	13,768	NR	NR	NR	5-yr CrSurv = 77.70% (70.80% −85.10%)	NR	NR	5-yr CrSurv = 90.20 (89.60–90.80)
Condon, 2016 [64]	NT	2007–2012	NT Cancer Registry	NR	196	1087	AAIR = 90.50	NR	Excess HR = 3.10 (2.00–5.20)	NR	AAIR = 104.50	NR	NR
Moore, 2016 [6]	QLD	1998–2004	QLD Cancer Registry	≥18	110	105	NR	NR	aHR = 1.52 (0.81- 2.84)	NR	NR	NR	NR
Tervonen, 2017 [65]	NSW	2000–2008	NSW Cancer Registry	All ages	331	32,754	NR	NR	aHR = 1.62 (1.22–2.16)	NR	NR	NR	NR
Read, 2020 [66]	NT	2001–2010	NT Cancer Registry	All ages	100	396	AAIR = 70.0	NR	NR	5-yr CrSurv = 70.6	NR	NR	5-yr CrSurv = 87.3
Dasgupta, 2022 [67]	QLD, WA, NT, and NSW	2005–2016	QLD, WA, NT, and NSW Cancer Registry	15–84	1677	NR	NR	NR	NR	5-yr CrSurv = 75.60 (73.50–77.80)	NR	NR	5-yr CrSurv = 88.4 (88.10–88.70)
Dasgupta, 2022 [68]	QLD, WA, NT, and NSW	2005–2016	QLD, WA, NT, and NSW Cancer Registry	15–84	1677	NR	NR	NR	5-year probability = 0.20 (0.10–0.20)	NR	NR	NR	NR
Cramb, 2023 [69]	QLD	1997–2016	QLD Cancer Registry	20–89	226	30,871	NR	NR	NR	5-yr CrSurv = 85.30 (82.90–87.80)	NR	NR	5-yr CrSurv = 91.0 (90.60–91.50)
**New Zealand**													
Campbell, 2015 [14]	National	1998–2010	Quality Audit Database	All ages	1428	9024	NR	NR	aHR = 1.46 (1.22–1.75)	5-yr CrSurv = 87.40	NR	NR	5-yr CrSurv = 90.70
Seneviratne, 2015 [19]	Waikato	1999–2012	Waikato Cancer Registry	NR	419	2260	NR	NR	aHR = 1.40 (1.09–1.81)	5-yr CrSurv = 76.10	NR	NR	5-yr CrSurv = 86.80
Seneviratne, 2015 [70]	Waikato	2002–2010	Waikato Cancer Registry	NR	133	732	NR	NR	AOR = 0.85 (0.49–1.48)	NR	NR	NR	NR
Teng, 2016 [71]	National	2006–2011	New Zealand Mortality and Cancer Registry	NR	NR	NR	AAIR = 140.1	AAMR = 19.60	NR	NR	AAIR = 95.80	AAMR = 18.20	NR
Lawrenson, 2017 [72]	Waikato and Auckland	2000–2013	Waikato and Auckland Cancer Registry	All ages	891	7576	NR	NR	aHR = 1.52 (1.06–2.18)	5-yr CrSurv = 94.20	NR	NR	5-yr CrSurv = 96.50
Ballantine, 2018 [33]	National	2000–2009	New Zealand Cancer Registry	25–29	30	67	IR/1,000,000 = 79.00 (51.00–107.00)	NR	NR	5-yr CrSurv = 47.00 (25.00–66.00)	IR/1,000,000 = 36.00 (27.00–44.00)	NR	5-yr CrSurv = 70.00 (56.0–100.0)
Tin, 2018 [13]	Waikato and Auckland	2000–2014	Auckland and Waikato Cancer Registry	All ages	1283	11,489	NR	NR	aHR = 1.70 (1.50–2.00)	NR	NR	NR	NR
Gurney, 2020 [73]	National	2007–2016	New Zealand Cancer Registry	≥18	NR	NR	NR	NR	aHR = 1.40 (1.20–1.50)	5-yr CrSurv = 86.60	NR	NR	5-yr CrSurv = 89.00
**Canada**													
Nishri, 2015 [74]	Ontario	1992–2001	Ontario Cancer Registry	30–74	3942	NR	NR	NR	aHR = 1.30 (1.00–1.76)	5-yr AdSurv = 74.40 (68.80–80.40)	NR	NR	5-yr AdSurv = 83.70 (83.30 -84.00)
Decker, 2016 [75]	Manitoba	2004–2008	Manitoba Cancer Registry	≥15	131	3914	AAIR = 99.60	AAMR = 22.70	NR	NR	AAIR = 135.00	AAMR = 30.00	NR
Withrow, 2017 [76]	National	1992–2009	Canadian Cancer Registry	45–90	580	29,265	NR	NR	NR	5-yr AdSurv = 75.30 (68.80–82.40)	NR	NR	5-yr AdSurv = 87.00 (86.00–88.00)
Mazereeuw, 2018 [77]	National	1992–2009	Canadian Cancer Registry	≥25	165	NR	AAIR = 165.20	NR	NR	5-yr AdSurv = 79.60 (68.00–93.20)	AAIR = 146.00	NR	5-yr AdSurv = 87.00 (86.00–88.00)
Wilkinson, 2024 [29]	National	2006 and 2011	Canadian Census Health and Environment Cohort database	All ages	1335	39,695	AAIR = 117.6 0 (131.30-135.70)	AAMR = 24.20 (22.00-26.60)	NR	AAIR = 135.7 (134.3-137.2	NR	AAMR = 22.50 (22.20-23.00)	NR
**Peru**													
Tamayo, 2018 [27]	National	2010–2015	Clinical records, Instituto Nacional de Enfermedades Neoplásicas	All ages	303	NR	NR	NR	NR	PropAlive = 79.00	NR	NR	NR
**Brazil**													
Borges, 2022 [31]	Acre	2000–2012	Goiania Cancer Registry	All ages	3	NR	SIR = 0.12 (0.11–0.14)	NR	NR	NR	NR	NR	NR
Guimarães Ribeiro, A, 2023 [28]	São Paulo	2001–15	Informatics Department of the Unified Health System (DATASUS)-Information System on Mortality	All ages	4	13,348	NR	AAMR = 3.80 (0.00-7.60)	NR	NR	NR	AAMR = 17.20 (16.90-17.50)	NR
**Colombia**													
Suescun, 2025 [30]	National	2011 - 2022	National Administrative Department of Statistics (DANE)	All ages	4938	465,668	NR	AAMR = 1.5 (1.3-1.7)	NR	NR	NR	AAMR = 9.1 (9.0-9.1)	NR

Abbreviations: 5-yr AdSurv: 5-year adjusted survival rate; 5-yr CrSurv: 5-year crude survival rate; AAIR: age-adjusted breast cancer incidence rate/100,000; AAMR: age-adjusted breast cancer breast cancer mortality rate/100,000; APC: Annual Percent Change; aHR: adjusted hazard ratio; CDC: Centers for Disease Prevention and Control; HR: hazard ratio; IR: breast cancer incidence rate; MR: breast cancer mortality rate; NPCR: National Population Cancer Registry; NSW: New South Wales; NR: not reported; NT: Northern Territory; PropAlive: proportion alive; QLD: Queensland; SD: standard deviation; SEER: Survival Epidemiology and End Result; SIR: standardized breast cancer incidence rate; USA: United States of America; WA: Western Australia.

a Reference: non-Indigenous women.

### 3.3 Incidence rates

**Fig. 2:**
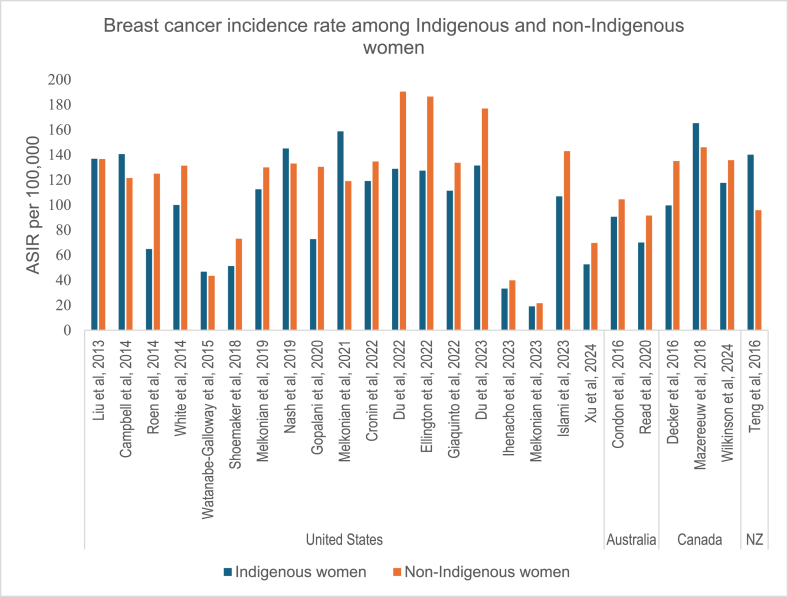
Breast cancer incidence rates per 100,000 among Indigenous and non-Indigenous women, 2013–2025. Abbreviations: ASIR-Age standardized incidence rate; NZ-New Zealand.

Of the 61 studies included in the analysis, 28 reported breast cancer incidence rates; 27 studies included both Indigenous women and non-Indigenous women, while one study included only Indigenous women. Geographically, 20 studies were conducted in the United States, three in Canada, two studies each in New Zealand and Australia, and one study in Brazil. The methods used to determine incidence rates varied. For example, of the 25 studies that reported the age-adjusted breast cancer incidence rates per 100,000, 18 used the 2000 United States standard population for age standardization, while seven used alternative standardization methods. Additionally, several studies [[33], [44], [50], [58], [61]] included only adolescents and young adults. The age-adjusted breast cancer incidence rates among Indigenous people ranged from 19.0 per 100,000 in the United States [58] to 165.2 per 100,000 in Canada [77]. Similarly, the age-adjusted breast cancer incidence rates among non-Indigenous people ranged from 21.5 to 190.4 per 100,000 in the United States [58] (Table 1) (Fig. 2).

### 3.4 Mortality rate

Of the 61 studies reviewed, 13 reported breast cancer mortality rates in both Indigenous and non-Indigenous women, including eight studies conducted in the United States, two in Canada, and one each conducted in Brazil, Colombia, and New Zealand. Twelve studies presented age-adjusted breast cancer mortality rates per 100,000 person-years. Of these, seven studies used the 2000 United States standard population for age standardization, one used the World Health Organization world standard population, and four studies used alternative standardization methods. The age-adjusted breast cancer mortality rates for Indigenous women per 100,000 person-years varied from 1.5 in Colombia, including all age groups [30] to 29.6 in the United States, including only those above 35 years of age [34]. By contrast, mortality rates for non-Indigenous women varied from 9.1 per 100,000 in Colombia, including all age groups [30] to 47.5 in the United States for those older than 35 years of age [34] (Table 1).

### 3.5 Survival rates

**Fig. 3:**
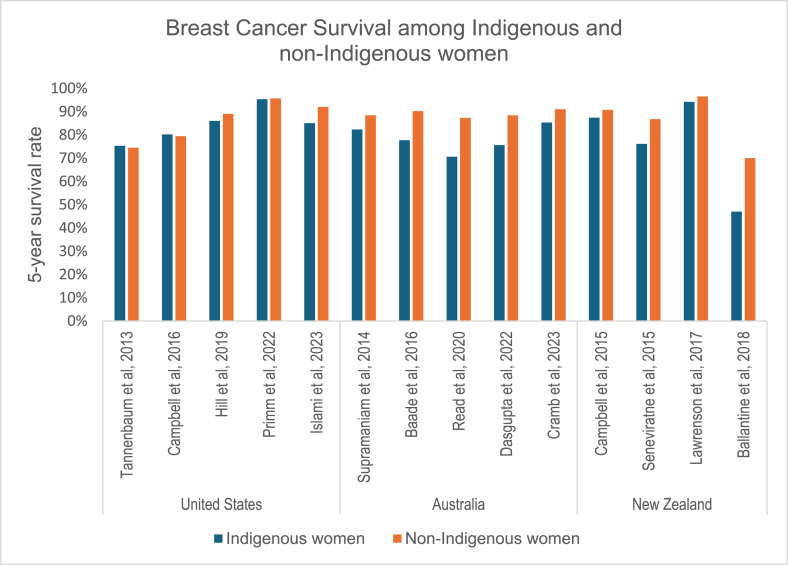
Breast Cancer Survival among Indigenous and non-Indigenous women, 2013–2025.

Twenty-two of the 61 studies reported breast cancer survival rates. Of these, 18 reported results for both Indigenous and non-Indigenous women, while four focused solely on Indigenous women. The 22 studies were conducted in the United States (n = 8), New Zealand (n = 5), Australia (n = 5), Canada (n = 3), and Peru (n = 1). Thirteen of these studies reported breast cancer–specific survival, and nine reported overall breast cancer survival using various measures of survival rates; 16 of the 22 studies used the five-year crude survival rate, whereas six studies used alternative measurements. All sixteen studies reported the five-year crude survival rate among Indigenous women, while 14 also reported the five-year survival rate among non-Indigenous women. The five-year crude breast cancer survival rate determined for Indigenous women ranged from 47% in New Zealand among 25–29 year olds [33] to 95.3% in the United States among adults 20 years of age and above [54]. By contrast, the five-year survival rate for non-Indigenous women in New Zealand ranged from 70% among 25–29 year-olds [33] to 96.5% [72] (Table 1) (Fig. 3).

### 3.6 Breast cancer patient hazard of mortality: Indigenous compared to non-Indigenous women

Twenty-one of the 61 studies reported estimates of the hazard of mortality among Indigenous women with breast cancer using various measurement units. Of these, 17 studies featured adjusted hazard ratios (aHRs), while others used excess hazard ratios, adjusted odds ratios, and unadjusted HRs (each n = 1). Among studies reporting aHRs for breast cancer mortality, eight were conducted in the United States, five in New Zealand, three in Australia, and one in Canada. In two studies performed in the United States that included non-Indigenous women as the reference, the aHRs for breast cancer mortality ranged from 1.03 to 4.65 [43] (Table 1) indicating lower breast cancer survival rates among Indigenous compared to non-Indigenous women.

### 3.7 Pooled adjusted hazard ratio for breast cancer mortality

**Fig. 4:**
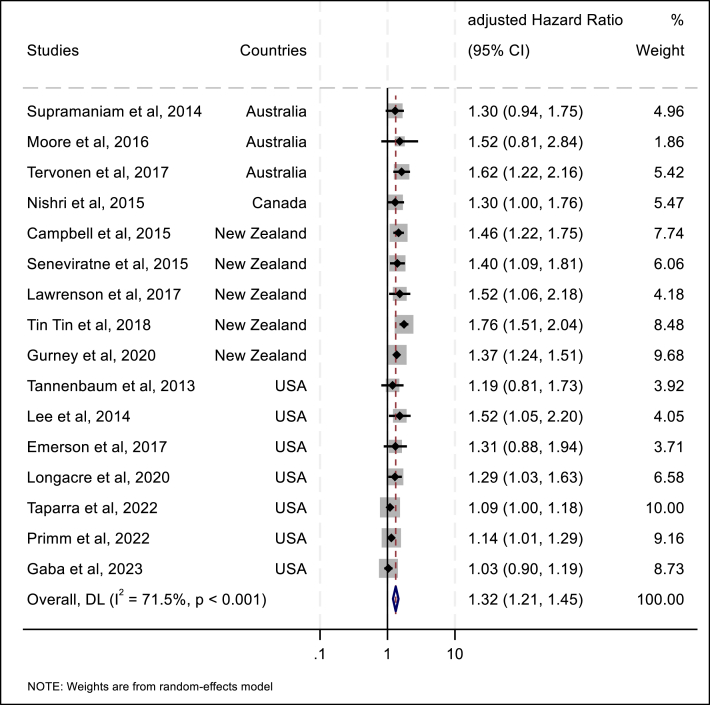
Forest plot of aHRs of breast cancer determined for Indigenous women. Abbreviations: USA-United States; DL-DerSimonian and Laird.

In our DerSimonian and Laird random-effects meta-analysis, the global pooled aHR for breast cancer mortality was 1.32 (95% CI: 1.21–1.45) among Indigenous women compared to non-Indigenous women. The pooled estimate of Cochran's *Q*-test and *I*^2^ indicated significant between-study heterogeneity at *p <* 0.001 and *I*^2^ = 71.5%. Egger's test, with a *p*-value of 0.131, suggesting no publication bias among the included studies. The results of the meta-analysis are presented in a forest plot (Fig. 4).

### 3.8 Subgroup analysis of adjusted hazard ratio for breast cancer mortality

A subgroup analysis by region revealed a pooled aHR for breast cancer mortality of 1.48 (95% CI: 1.37–1.61) in Oceania and 1.13 (95% CI: 1.06–1.20) in North America. A subgroup analysis based on publication year indicated a pooled aHR of 1.42 (95% CI: 1.29–1.56) for studies published from 2013 to 2017, and 1.25 (95% CI: 1.08–1.45) for those published from 2018 to 2023. Furthermore, a subgroup analysis based on participant age revealed pooled aHRs for breast cancer mortality of 1.57 (95% CI: 1.39–1.77) among studies covering all age groups and 1.21 (95% CI: 1.11–1.32) among studies focused on participants aged 15 years or older (Table 2).

**Table 2:** Subgroup analysis of adjusted hazard ratios for breast cancer mortality among Indigenous versus non-Indigenous women, 2013–2025.

Subgroup variables	aHR	95% CI		Heterogeneity		No. Articles
		LCI	UCI	*I*^2^*(%)*	*p-v*alue	
Total	1.32	1.21	1.45	71.5	<0.001	16
**Region**						
Oceania	1.48	1.37	1.61	19.6	0.274	8
North America	1.13	1.06	1.20	8.3	0.366	8
**Publication year**						
2013-2017	1.42	1.29	1.56	0.00	0.968	10
2018-2025	1.25	1.08	1.45	88.1	<0.001	6
**Participant Age**						
All ages	1.57	1.39	1.77	0.00	0.479	6
≥15 years	1.21	1.11	1.32	55.0	0.018	10

Abbreviations: CI-confidence interval; LCI-lower confidence interval; UCI-upper confidence interval.

### 3.9 Meta-regression of aHRs for breast cancer mortality

We conducted a meta-regression analysis of the findings presented in the 61 studies to identify potential sources of their significant heterogeneity. In the multivariable meta-regression, participant age and the geographic region covered by the study were identified as significant sources of heterogeneity. Together with the year of publication, these variables explained 98% of the between-study variation (i.e., *I*^*2*^ and *τ*^*2*^ were substantially reduced) (Table S4).

### 3.10 Sensitivity analysis of aHRs for breast cancer mortality

Sensitivity analysis revealed that pooled aHRs for breast cancer mortality determined after omission of single studies ranged from 1.40 (95% CI: 1.31–1.51) to 1.56 (95% CI: 1.42–1.71) for studies conducted in Oceania and from 1.12 (95% CI: 1.05–1.18) to 1.16 (95% CI: 1.07–1.22) for studies performed in North America (Table S5). Furthermore, sensitivity analysis revealed that no single study had a significant impact on the pooled estimates; all pooled estimates derived from the omission of a single study remained within the CI of the overall calculation (Table S5).

## 4 Discussion

This systematic review and meta-analysis of 61 studies published since 2013 comprehensively synthesized breast cancer incidence, mortality, and survival rates among Indigenous women. Overall, despite lower incidence rates, we found that Indigenous women experienced higher mortality and lower survival rates than non-Indigenous women worldwide. This disparity might be associated with a combination of genetic, socioeconomic, healthcare accessibility, breast cancer screening participation, and cultural factors.

All 61 studies reported that breast cancer incidence rates were lower among Indigenous than non-Indigenous women. As breast cancer incidence rates among Indigenous women are not reported globally, it is difficult to compare our findings to previously published data. However, our findings do align with government reports from some countries, such as the United States, where the age-adjusted breast cancer incidence rate in 2022 among American Indians and Alaska Natives was reported to be 117.2 per 100,000, a value lower than that determined for non-Hispanic Whites (139.2 per 100,000) [78]. Similarly, between 2009 and 2013, the age-adjusted incidence rate of breast cancer among Indigenous Australians was 98.8 per 100,000, a value lower than that determined for non-Indigenous Australians during the same period (111.7 per 100,000) [12]. The lower incidence rate of breast cancer among Indigenous compared to non-Indigenous women might be a data-related issue, for example, under-reporting and misclassification of Indigenous status in medical records and cancer registry databases. This difference might also reflect Indigenous women's limited access to healthcare services. The lower incidence of breast cancer among Indigenous women might also be related to specific protective factors, such as higher fertility rates for specific hormone-related breast cancers. For instance, the average birth rate for Aboriginal and Torres Strait Islander women in Australia was 2.2 births per woman, compared to 1.5 births per woman for all Australians in 2023 [79].

Our findings also indicated an increase in breast cancer incidence between the years 2013 and 2025 among both Indigenous and non-Indigenous women worldwide. This finding is consistent with global predictions of a 54.7% increase in breast cancer incidence from 2022 to 2050 [80]. Additionally, breast cancer incidence rates in Australia increased from 134.7 per 100,000 in 2011 to 147.8 per 100,000 in 2021 [81], and from 94.2 per 100,000 in 2011 to 95.7 per 100,000 in 2021 in New Zealand [82]. The global increase in the incidence of breast cancer may be related to several factors, including Westernized lifestyle changes and environmental exposures (for example, changes in reproductive patterns, dietary habits, and exposure to exogenous estrogen), and a higher detection rate through population-based screening programs [83,84].

Our findings revealed that the hazard of breast cancer mortality among women diagnosed with the disease was 32% higher among Indigenous than non-Indigenous women. This finding was supported by the lower five-year breast cancer survival rate observed among Indigenous women, ranging from 47% to 95% among Indigenous women and 70% to 96% among non-Indigenous women. This difference in mortality is associated with risk factors encountered by Indigenous women, including poor access to treatment [15,18], advanced-stage diagnosis [54], insufficient diagnostic and treatment infrastructure [85], and comorbidities [15].

We also determined that Indigenous women experienced different hazard of mortality in different regions of the world and over different periods. For instance, the aHR for breast cancer mortality among Indigenous women was pooled to be 42% based on the studies published between 2013 and 2017; this value was reduced to 25% using data from studies published between 2018 and 2025. This difference might be associated with improved early breast cancer detection as a result of national screening programs. For example, the participation rate in breast cancer screening among Indigenous women in Australia increased from 29% in 2010–2011 to 36% in 2022–2023 [86]. Additionally, while the hazard of breast cancer mortality among Indigenous women in Oceania was 48%, it was 13% among Indigenous women in North America during the same period. Differences were also observed in government reports, documenting five-year breast cancer survival rates of 88% among American Indian/Alaska Native women [87] and 81% among Indigenous Australians in 2007–2014 [12]. This difference might be associated with the higher breast cancer screening rates among American Indian/Alaska Native women (62%) [87] compared with Indigenous Australians (36%) reported specifically for the years 2022–2023 [86]. While the comparatively low counts in Indigenous populations reduce the precision of these estimates, the differences may still be clinically significant and/or socio-culturally meaningful. Therefore, interventions aimed at improving breast cancer screening participation rates among Indigenous women are most likely essential for improving breast cancer survival outcomes. However, further research will be needed to understand the disparity in survival using a broader time frame, such as the 10-year survival rate, as this disparity might increase.

To the best of our knowledge, this study is the first to provide a comprehensive synthesis of the epidemiological evidence of the burden of breast cancer among Indigenous women worldwide. We believe that our findings will help fill gaps in our understanding of the burden of breast cancer among Indigenous women worldwide. Our review also highlights a need for an improved focus on Indigenous populations in databases containing longitudinal data, as well as enhanced recording and reporting of breast cancer incidence and outcomes among Indigenous women. Moreover, our findings revealed that there are very few studies that focused specifically on understanding the burden of breast cancer among Indigenous peoples in some areas of the world. In our case, only four eligible studies included participants from Latin America, and none from Africa or Asia. This is significant, given that approximately 80% of the world's Indigenous peoples reside in these regions [10]. This lapse may be related to poor data access as well as Indigenous misclassification and/or inadequate identification, all of which need to be improved in the future.

This review has several limitations that should be considered when interpreting the results. First, the review was restricted to studies published in English, thereby limiting the generalizability of the findings to non-English-speaking populations. Second, this review was limited to studies published since 2013 and thus focuses on the most recent evidence. Third, although the search captured peer-reviewed articles from multiple databases, it did not include grey literature. Unpublished reports of breast cancer burden among Indigenous women might have been excluded, potentially leading to an over- or underestimation of the pooled effect. Finally, although the studies included in this review exhibited high heterogeneity, we addressed this issue through subgroup analysis, sensitivity analysis, and meta-regression.

## 5 Conclusion

This review analyzed results presented in 61 published studies, summarized the global breast cancer incidence, mortality, and survival rates among Indigenous women, and compared them with those determined for non-Indigenous women. Overall, Indigenous women were found to have poorer survival outcomes and a higher hazard of mortality than non-Indigenous women, despite a lower incidence rate of breast cancer. Although the hazard of mortality has declined over time and the five-year breast cancer survival rate in this population has improved, disparities in breast cancer incidence, mortality, and survival between Indigenous and non-Indigenous women persist. An international commitment to co-designing early detection strategies with Indigenous communities, including approaches to increase breast screening participation, developing more efficient referral care pathways, and improving access to comprehensive treatment, will be required to address disparities in breast cancer survival.

## CRediT authorship contribution statement

**Halijah Brewster:** Writing – review & editing, Writing – original draft, Visualization, Project administration, Methodology, Investigation, Formal analysis, Data curation, Conceptualization. **Tsegaw Amare Baykeda:** Writing – review & editing, Writing – original draft, Visualization, Validation, Software, Project administration, Methodology, Investigation, Formal analysis, Data curation. **Sewunet Admasu Belachew:** Writing – review & editing, Validation, Supervision, Project administration, Methodology, Conceptualization. **Valerie McCormack:** Writing – review & editing. **Miranda Fidler-Benaoudia:** Writing – review & editing, Conceptualization. **Gail Garvey:** Writing – review & editing, Supervision, Methodology, Funding acquisition, Conceptualization.

## Ethical approval

Not applicable due to aggregated data having been extracted from publicly available and published peer-reviewed journal articles.

## Data availability and materials

All data reported in this study were extracted from publicly available published articles. All pertinent information from the included articles has been included within the main manuscript and supplementary materials.

## Funding

GG was supported by the 10.13039/501100000925NHMRC Investigator Grant (2034453). 10.13039/501100016226HB has been funded by a post-graduate research scholarship from 10.13039/501100001794The University of Queensland. The funders had no direct or indirect involvement in the study's design, data analysis, interpretation, or in the decision to submit the manuscript for publication.

## Conflict of interest statement

The authors declare that they have no conflicts of interest.
