# Supplementary material for: Global breast cancer incidence, mortality, and survival among indigenous women: A systematic review and meta-analysis
Source: Breast. 2026 Feb 26;86:104742. doi: 10.1016/j.breast.2026.104742 (PMC12972959; doi:10.1016/j.breast.2026.104742)
Supplement: Multimedia component 2 [file mmc2.docx]

**Table S2: Search terms for breast cancer incidence, mortality, and survival among Indigenous populations worldwide**

**Search date: 9/06/2025**

| **Search Filters/results** | **Searching for** | **Search terms** |
| --- | --- | --- |
| #1 *Filters applied:*Humans, English*.* | **Breast Cancer** | ((("breast"[All Fields]) OR breast [MeSH Terms] AND ((neoplasms [MeSH Terms]) OR (cancer*[All Fields] OR Carcinoma [All fields] OR Tumour [All fields] OR Malignan* [All Fields])))) |
| #2 *Filters applied:*Humans, English*.* | **Incidence, Mortality, and Survival** | (Incidence [MeSH] OR incidence [All Fields] OR Mortality [MeSH] OR mortality [All Fields] OR Death [All fields] OR fatality [All fields] OR prognosis [MeSH] OR Survival [MeSH] OR survival [All Fields] OR "life table" [All Fields] OR “hazard ratio" [All Fields]) |
| #3 *Filters applied:*Humans, English*.* | **Indigenous**  **Populations** | ((("First Nation*"[All Fields] OR ("american indian or alaska native"[MeSH Terms]) OR ("american indian or alaska native"[All Fields]) OR ("american native continental ancestry group"[All Fields]) OR ("oceanic ancestry group"[All Fields]) OR "australian aboriginal and torres strait islander peoples"[MeSH Terms] OR ("indigen*"[All Fields]) OR ("indians, north american"[MeSH Terms]) OR ("north american indians"[All Fields]) OR ("aborigin*"[All Fields]) OR native 3 NEAR (indian* or american* or canadian*) OR ("inuit"[MeSH Terms]) OR ("inuit*"[All Fields]) OR ("metis"[All Fields]) OR ("pacific islander*"[All Fields]) OR ("samoa*"[All Fields]) OR ("american samoa"[MeSH Terms]) OR ("american samoa"[All Fields]) OR ("pacific islands"[MeSH Terms]) OR "pacific island people"[MeSH Terms] OR ("pacific islands"[All Fields]) OR (Polynesia [MeSH Terms]) OR (polynesia[All Fields]) OR "maori people"[MeSH Terms] OR (maori*[All Fields]) OR "native hawaiian or other pacific islander"[MeSH Terms] OR ("Native Hawaiian*"[All Fields]) OR (Kanaka Maoli*[All Fields]) OR ("Aborigin*"[All Fields]) OR ("Torres Strait Islander*"[All Fields]) OR ("haida"[All Fields]) OR ("cree"[All Fields]) OR ("ojibwe"[All Fields]) OR ("anishnawbe"[All Fields]) OR ("anishinaabe*"[All Fields]) OR ("mohawk*"[All Fields]) OR ("dene"[All Fields]) OR ("algonquin*"[All Fields]) OR ("mississauga*"[All Fields]) OR ("seneca"[All Fields])))) OR "African continental ancestry group" OR "Asian continental ancestry group"[All Fields] OR Indigen*/Africa OR Indigen*/Asia OR Indigen*/Europe OR tribe OR tribal OR "scheduled ~tribe*" OR indigen*/Indian OR "native ~people" OR "first people" OR Indigen*/Brazil OR Aborigin*/Brazil OR Mapuche[All Fields] |
| Search result for **PubMed** *Filters applied:*Humans, English last ten years*.* | **#1 AND #2**  **AND #3 =** **927 results** | (((Incidence [MeSH] OR incidence [All Fields] OR Mortality [MeSH] OR mortality [All Fields] OR Death [All fields] OR fatality [All fields] OR prognosis [MeSH] OR Survival [MeSH] OR survival [All Fields] OR "life table" [All Fields] OR "hazard ratio" [All Fields])) AND (((("breast"[All Fields]) OR breast [MeSH Terms] AND ((neoplasms [MeSH Terms]) OR (cancer*[All Fields] OR Carcinoma [All fields] OR Tumour [All fields] OR Malignan* [All Fields])))))) AND (((("First Nation*"[All Fields] OR ("american indian or alaska native"[MeSH Terms]) OR ("american indian or alaska native"[All Fields]) OR ("american native continental ancestry group"[All Fields]) OR ("oceanic ancestry group"[All Fields]) OR "australian aboriginal and torres strait islander peoples"[MeSH Terms] OR ("indigen*"[All Fields]) OR ("indians, north american"[MeSH Terms]) OR ("north american indians"[All Fields]) OR ("aborigin*"[All Fields]) OR native 3 NEAR (indian* or american* or canadian*) OR ("inuit"[MeSH Terms]) OR ("inuit*"[All Fields]) OR ("metis"[All Fields]) OR ("pacific islander*"[All Fields]) OR ("samoa*"[All Fields]) OR ("american samoa"[MeSH Terms]) OR ("american samoa"[All Fields]) OR ("pacific islands"[MeSH Terms]) OR "pacific island people"[MeSH Terms] OR ("pacific islands"[All Fields]) OR (Polynesia [MeSH Terms]) OR (polynesia[All Fields]) OR "maori people"[MeSH Terms] OR (maori*[All Fields]) OR "native hawaiian or other pacific islander"[MeSH Terms] OR ("Native Hawaiian*"[All Fields]) OR (Kanaka Maoli*[All Fields]) OR ("Aborigin*"[All Fields]) OR ("Torres Strait Islander*"[All Fields]) OR ("haida"[All Fields]) OR ("cree"[All Fields]) OR ("ojibwe"[All Fields]) OR ("anishnawbe"[All Fields]) OR ("anishinaabe*"[All Fields]) OR ("mohawk*"[All Fields]) OR ("dene"[All Fields]) OR ("algonquin*"[All Fields]) OR ("mississauga*"[All Fields]) OR ("seneca"[All Fields])))) OR "African continental ancestry group" OR "Asian continental ancestry group"[All Fields] OR Indigen*/Africa OR Indigen*/Asia OR Indigen*/Europe OR tribe OR tribal OR "scheduled ~tribe*" OR indigen*/Indian OR "native ~people" OR "first people" OR Indigen*/Brazil OR Aborigin*/Brazil OR Mapuche[All Fields]) |
| **Embase** *Filters applied:*Humans, English, last ten years*.* | **2,090** | (((Incidence/exp OR incidence OR Mortality/exp OR mortality OR Death OR fatality OR prognosis/exp OR Survival/exp OR survival OR 'life table' OR 'hazard ratio')) AND ((((breast) OR breast/exp AND ((neoplasms/exp) OR (cancer* OR Carcinoma OR Tumour OR Malignan*)))))) AND (((('First Nation*' OR ('american indian or alaska native'/exp) OR ('american indian or alaska native') OR ('american native continental ancestry group') OR ('oceanic ancestry group') OR 'australian aboriginal and torres strait islander peoples'/exp OR (indigen*) OR ('indians, north american'/exp) OR ('north american indians') OR (aborigin*) OR 'native 3 NEAR' (indian* OR american* OR canadian* ) OR (inuit/exp) OR (inuit*) OR (metis) OR ('pacific islander*') OR (samoa*) OR ('american samoa'/exp) OR ('american samoa') OR ('pacific islands'/exp) OR 'pacific island people'/exp OR ('pacific islands') OR (Polynesia/exp) OR (polynesia) OR 'maori people'/exp OR (maori*) OR 'native hawaiian or other pacific islander'/exp OR ('Native Hawaiian*') OR ('Kanaka Maoli*') OR (Aborigin*) OR ('Torres Strait Islander*') OR (haida) OR (cree) OR (ojibwe) OR (anishnawbe) OR (anishinaabe*) OR (mohawk*) OR (dene) OR (algonquin*) OR (mississauga*) OR (seneca)))) OR 'African continental ancestry group' OR 'Asian continental ancestry group' OR Indigen*/de OR Indigen*/de OR Indigen*/de OR tribe OR tribal OR 'scheduled tribe*' OR indigen*/de OR 'native people' OR 'first people' OR Indigen*/de OR Aborigin*/de OR Mapuche) |
| **CINAHL** *Filters applied:*last ten years, English*.* | **355** | ((((MH Incidence+) OR incidence OR (MH Mortality+) OR mortality OR Death OR fatality OR (MH prognosis+) OR (MH Survival+) OR survival OR "life table" OR "hazard ratio")) AND ((((breast) OR (MH breast+) AND (((MH neoplasms+)) OR (cancer* OR Carcinoma OR Tumour OR Malignan*)))))) AND (((("First Nation*" OR ((MH "american indian or alaska native+")) OR ("american indian or alaska native") OR ("american native continental ancestry group") OR ("oceanic ancestry group") OR (MH "australian aboriginal and torres strait islander peoples+") OR (indigen*) OR ((MH "indians, north american+")) OR ("north american indians") OR (aborigin*) OR "native 3 NEAR" (indian* OR american* OR canadian* ) OR ((MH inuit+)) OR (inuit*) OR (metis) OR ("pacific islander*") OR (samoa*) OR ((MH "american samoa+")) OR ("american samoa") OR ((MH "pacific islands+")) OR (MH "pacific island people+") OR ("pacific islands") OR ((MH Polynesia+)) OR (polynesia) OR (MH "maori people+") OR (maori*) OR (MH "native hawaiian or other pacific islander+") OR ("Native Hawaiian*") OR ("Kanaka Maoli*") OR (Aborigin*) OR ("Torres Strait Islander*") OR (haida) OR (cree) OR (ojibwe) OR (anishnawbe) OR (anishinaabe*) OR (mohawk*) OR (dene) OR (algonquin*) OR (mississauga*) OR (seneca)))) OR "African continental ancestry group" OR "Asian continental ancestry group" OR (MH Indigen*) OR (MH Indigen*) OR (MH Indigen*) OR tribe OR tribal OR "scheduled ~tribe*" OR (MH indigen*) OR "native ~people" OR "first people" OR (MH Indigen*) OR (MH Aborigin*) OR Mapuche) |
| **Web of science** *refined by Language: English and document type: Article* | 753 | (((ALL=breast) OR ALL=breast AND ((ALL=neoplasms) OR (ALL=cancer* OR ALL=Carcinoma OR ALL=Tumour OR ALL=Malignan*)))) AND (ALL=Incidence OR ALL=incidence OR ALL=Mortality OR ALL=mortality OR ALL=Death OR ALL=fatality OR ALL=prognosis OR ALL=Survival OR ALL=survival OR ALL="life table" OR ALL="hazard ratio") AND (ALL="Indigenous Peoples" OR ALL="Indigenous Canadians" OR ALL="American Indian or Alaska Native" OR ALL=Oceanians OR ALL="Health Services, Indigenous" OR ALL="Indians, North American" OR ALL="Maori People" OR ALL="Australian Aboriginal and Torres Strait Islander Peoples" OR ALL=Inuit OR ALL="American Samoa" OR ALL="Pacific Islands" OR ALL=Micronesia OR ALL=Samoa OR ALL=Polynesia OR ALL=(Native AND (Indian* OR American* OR Canadian*)) OR ALL="First Nation*" OR ALL=Metis OR ALL="Maori*" OR ALL="Native Hawaiian*" OR ALL="Kanaka Maoli*" OR ALL=Aborigine* OR ALL="Torres Strait Islander*" OR ALL=Haida OR ALL=Cree OR ALL=Ojibwe OR ALL=Anishnawbe OR ALL=Anishinaabe* OR ALL=Mohawk OR ALL=Dene OR ALL=Algonquin* OR ALL=Mississauga* OR ALL=Seneca OR ALL="African continental ancestry group" OR ALL="Asian continental ancestry group" OR ALL=Indigen* OR ALL=Indigen* OR ALL=Indigen* OR ALL= tribe OR ALL=tribal OR ALL="scheduled ~tribe*" OR ALL=indigen* OR ALL="native ~people" OR ALL="first people" OR ALL=Indigen* OR ALL=Aborigin* OR ALL=Mapuche) |

**Inclusion and exclusion criteria**

1. Included Indigenous women with breast cancer; 2) included a comparative non-Indigenous female population with breast cancer; 3) assessed and **reported at least one of the three main outcomes**: incidence, mortality, or survival after a diagnosis of breast cancer; 4) reported data from population or hospital-based registries, published in **English** and 5) was the most recent data available:

- If more than one paper from a jurisdiction was eligible, we adopted the following selection hierarchy 1) best data source for case ascertainment; 2) used the World Standard Population (WSP) to calculate age-adjusted statistics, as this was the most commonly used measure across jurisdictions or reported the most recent data.
- Studies that repeated previously published results were excluded, as were those that combined results over extended periods i.e., more than 10 years, since these were more likely to include data from periods when Indigenous cancer rates were underestimated. Exceptions were when these studies were the only results available for that country.

1. Articles were excluded if they were: 1) commentary pieces/editorials; 2) review papers; 3) randomised controlled trials; 4) methodology/protocol papers; 5) research dissertations; 7) qualitative studies/case studies; and 8) conference abstracts.

**Indigenous status**

We included papers that specified either an Indigenous population or a population that is recognised as ‘Indigenous’ using the UN guidelines, specifically peoples who identify and are identified as Indigenous, demonstrate historical continuity with pre-colonial societies, have distinct social, economic, or political systems, maintain distinct languages, cultures and beliefs and form non-dominant groups of society. (United Nations Inter-agency support group on Indigenous Peoples' issues 2014).

Non-Indigenous comparison populations varied across and within countries. Data sources included state, provincial or hospital-based cancer registries and national census data.
