# Supplementary material for: Global breast cancer incidence, mortality, and survival among indigenous women: A systematic review and meta-analysis
Source: Breast. 2026 Feb 26;86:104742. doi: 10.1016/j.breast.2026.104742 (PMC12972959; doi:10.1016/j.breast.2026.104742)
Supplement: Multimedia component 3 [file mmc3.docx]

Supporting information

Table S3: Risk of bias assessment using the Newcastle Ottawa Scale quality appraisal tool.

| **S/N** | **Studies** | **Selection** | **Comparability** | **Outcome/Exposure** |
| --- | --- | --- | --- | --- |
|  | DeGrubb, M | **** | ** | *** |
|  | Liu, L | **** | ** | ** |
|  | Tannenbaum, S | **** | ** | *** |
|  | Campbell, J | **** | ** | *** |
|  | Lee, D | **** | ** | * |
|  | Roen | **** | ** | *** |
|  | Supramaniam | **** | ** | ** |
|  | White, A | **** | * | *** |
|  | Campbell, I | ** | ** | *** |
|  | Nishri, E | **** | ** | *** |
|  | Seneviratne, S | **** | ** | ** |
|  | Seneviratne, S | **** | ** | *** |
|  | Watanabe- Galloway, S | **** | ** | *** |
|  | Baade, P | **** | ** | ** |
|  | Campbell, J | **** | ** | *** |
|  | Condon, J | **** | ** | ** |
|  | Decker, K | **** | ** | * |
|  | Moore, S | **** | ** | ** |
|  | Teng, A | **** | ** | ** |
|  | Emerson, M | **** | ** | *** |
|  | Khan, R | **** | * | ** |
|  | Lawrenson, R | **** | ** | *** |
|  | Tervonen, H | **** | ** | *** |
|  | Withrow, D | **** | ** | *** |
|  | Ballantine, K | **** | ** | *** |
|  | Mazereeuw, M | **** | ** | *** |
|  | Nash, S | **** | * | ** |
|  | Shoemaker, M | **** | ** | *** |
|  | Tamayo, L | **** | * | *** |
|  | Tin Tin, S | **** | * | ** |
|  | Hill, D | **** | ** | *** |
|  | Melkonian, S | **** | ** | *** |
|  | Nash, S | **** | ** | *** |
|  | Gopalani, S | **** | ** | *** |
|  | Gurney, J | **** | ** | *** |
|  | Longacre, C | **** | ** | ** |
|  | Read, D | **** | ** | ** |
|  | Hendrick, R | **** | ** | *** |
|  | Melkonian, S | **** | ** | *** |
|  | Borges, M | **** | ** | *** |
|  | Cronin, K | **** | ** | *** |
|  | Dasgupta, P | **** | ** | *** |
|  | Dasgupta, P | **** | ** | *** |
|  | Du, X | **** | ** | *** |
|  | Ellington, T | **** | ** | *** |
|  | Giaquinto, A | **** | ** | * |
|  | Primm, K | **** | ** | *** |
|  | Taparra, K | **** | ** | * |
|  | Cramb, S | **** | ** | * |
|  | Du, X | **** | ** | *** |
|  | Ellington, T | **** | ** | * |
|  | Gaba, A | ** | ** | ** |
|  | Ihenacho, U | **** | ** | *** |
|  | Melkonian, S | **** | ** | ** |
