# Supplementary material for: Global breast cancer incidence, mortality, and survival among indigenous women: A systematic review and meta-analysis
Source: Breast. 2026 Feb 26;86:104742. doi: 10.1016/j.breast.2026.104742 (PMC12972959; doi:10.1016/j.breast.2026.104742)
Supplement: Multimedia component 4 [file mmc4.docx]

**Table S4 - Meta-regression** **of adjusted hazard ratio**

| **Variable** | **aHR** | **Standard error** | ***z*** | ***p* > \|z\|** | **95% CI** |
| --- | --- | --- | --- | --- | --- |
| **Age group** | | | | | |
| All ages | Ref |  |  |  |  |
| ≥15 years | 0.84 | 0.06 | -2.30 | 0.021 | 0.72- 0.97 |
| **Publication year** | | | | | |
| 2013–2017 | Ref |  |  |  |  |
| 2018-2025 | 1.05 | 0.08 | 0.63 | 0.525 | 0.90-1.23 |
| **Region** | | | | | |
| Oceania | Ref |  |  |  |  |
| North America | 0.82 | 0.05 | -3.45 | 0.001 | 0.74-0.92 |
| τ^2^ | 0.000333 |  |  |  |  |
| *I*^2^ | 3.17% |  |  |  |  |
| *R*^2^ | 98.23 |  |  |  |  |
