# Supplementary material for: Global breast cancer incidence, mortality, and survival among indigenous women: A systematic review and meta-analysis
Source: Breast. 2026 Feb 26;86:104742. doi: 10.1016/j.breast.2026.104742 (PMC12972959; doi:10.1016/j.breast.2026.104742)
Supplement: Multimedia component 5 [file mmc5.docx]

**Table S5 - Sensitivity analysis of adjusted hazard ratios**

| **Study omitted** | **Estimate** | **LCI** | **UCI** |
| --- | --- | --- | --- |
| **Oceania** | **1.48** | **1.37** | **1.61** |
| Supramaniam et al, 2014 | 1.50 | 1.37 | 1.64 |
| Campbell et al, 2015 | 1.49 | 1.35 | 1.65 |
| Seneviratne et al, 2015 | 1.50 | 1.36 | 1.65 |
| Moore et al, 2016 | 1.49 | 1.36 | 1.63 |
| Lawrenson et al, 2017 | 1.48 | 1.35 | 1.63 |
| Tervonen et al, 2017 | 1.48 | 1.35 | 1.62 |
| Tin Tin et al, 2018 | 1.40 | 1.31 | 1.51 |
| Gurney et al, 2020 | 1.56 | 1.42 | 1.71 |
| **North America** | **1.13** | **1.06** | **1.20** |
| Tannenbaum et al, 2013 | 1.14 | 1.06 | 1.22 |
| Lee et al, 2014 | 1.12 | 1.05 | 1.18 |
| Nishri et al, 2015 | 1.12 | 1.05 | 1.20 |
| Emerson et al, 2017 | 1.13 | 1.06 | 1.21 |
| Longacre et al, 2020 | 1.12 | 1.05 | 1.19 |
| Primm et al, 2022 | 1.15 | 1.05 | 1.25 |
| Taparra et al, 2022 | 1.16 | 1.07 | 1.27 |
| Gaba et al, 2023 | 1.14 | 1.07 | 1.22 |
